# Nature-Based Environments for Health, Well-Being and Performance: A Hypothesis on Sport and Exercise Behavior

**DOI:** 10.3390/sports14090380

**Published:** 2026-09-01

**Authors:** Henrique Brito, Henrique Lopes, Eric Brymer, Duarte Araújo

**Affiliations:** 1 CIPER, Faculty of Human Kinetics, University of Lisbon, 1499-002 Cruz-Quebrada-Dafundo, Portugal; 2 Faculty of Health, Southern Cross University, Gold Coast, QLD 4225, Australia; 3 Manna Institute, University of New England, New England, NSW 2351, Australia

**Keywords:** athlete health, green places, movement expertise, prolonged engagement, skill adaptation

## Abstract

A fundamental concern for coaches and sport practitioners is developing athlete performance while preserving health and well-being. An emerging topic is that performing in nature-based environments may have more benefits than performing in typical built/indoor environments. However, to understand athlete performance and well-being, it is essential to recognize how performers accommodate (adapt) skills and resources under environmental constraints in pursuit of sport and exercise task goals. This article presents a narrative review to support a hypothesis on the sustained benefits of performing in nature-based environments, with health and well-being as necessary aspects for performance, grounded in the ecological dynamics theoretical framework. We start by discussing: (i) performance in nature-based and indoor environments underpinned by affordance perception; (ii) evidence for the role of nature-based environmental constraints on movement and psychological aspects; (iii) the hypothesis that prolonged engagement with nature-based environments during sport and exercise may promote adaptive skill development, health, and well-being, and may facilitate the transfer of these adaptations to performance in built indoor competitive settings; (iv) the distinct lived experiences (e.g., emotion, well-being) that emerge from exercising in nature-based and built indoor environments; and (v) suggestions on how to include nature-based constraints on sport and exercise training, and future research considerations. Overall, the reviewed theoretical and empirical evidence provides a rationale for the hypothesis that prolonged engagement with nature-based environments during sport and exercise may promote adaptive skill development, health, and well-being, with potential transfer of these adaptations to performance in built indoor settings. Future longitudinal and experimental studies are required to test this hypothesis and determine whether, and under what conditions, these proposed adaptations transfer to performance in built indoor sport settings.

## 1. Introduction

It is well known that performing in competitive sport may involve risk to health and well-being (comprising physical, mental, and social dimensions, and not merely the absence of disease or infirmity), possibly developing into injury or psychological distress. Athlete health and well-being are of great concern to coaches and sport practitioners, who discuss methods and develop strategies to decrease musculoskeletal injury [1], improve athlete emotional and mental well-being [2], and skill learning [3]. Over the last few decades, evidence has shown that exercising in nature can support health and well-being in the non-athlete population [4], with promising potential to enhance performance in recreational exercisers [5] (primarily behaving to meet sport and exercise task goals). The World Health Organization is clear in its endorsement of more urban green space for health and in prescribing nature-based exercise to the general population [6]; however, the crossover to competitive sport has not garnered much attention, apart from initial hypothesizing [7,8]. The ecological dynamics theoretical framework, with its focus on perception–action coupling as the basis for performance, has been at the forefront of understanding the potential benefits of nature-based exercise for enhancing performance and well-being in athletes [5,9]. This article is a narrative, hypothesis-generating review. The review aimed to integrate theoretical and preliminary empirical evidence on the role of nature-based environments in adaptive skill development, health, and well-being, and the extent to which these adaptations can transfer—that is, generalize to and enhance performance—in built indoor sport settings. Literature was identified through searches in the Web of Science databases for articles published up to May 2026. The search focused on peer-reviewed literature addressing nature-based sport and exercise, ecological dynamics, affordances, environmental constraints, motor learning, skill adaptation, health, and well-being. Studies were selected based on their conceptual and empirical relevance to these topics and synthesized to develop and support the proposed hypothesis. Next, the ecological dynamics theoretical framework is briefly summarized and proposed as an adequate theory to analyze sport and exercise performance, health, and well-being.

## 2. The Ecological Dynamics of Sport and Exercise Performance

The ecological dynamics theoretical framework considers the performer–environment system—the relation performers sustain with environmental circumstances in pursuit of task goals—as the relevant scale of analysis to understand performance, health, and well-being [10]. This is the ecological scale. The emphasis is on how performers regulate their body (including musculoskeletal, cardiovascular, and nervous systems) under changing environmental constraints to achieve sport and exercise goals [11]. Among key principles, Gibson’s [12] groundbreaking concepts of direct perception and affordances play a significant role in this framework. Structured information in the environment (e.g., the ambient patterns of energy, such as the optic array) specifies action possibilities (i.e., affordances) for the performer, allowing for direct perception. In sport and exercise, affordances are directly perceived by performers with specific skills and body characteristics, as well as goal-directed functions. For example, performing a push-up on the ground implies perceiving and acting upon push-up affordances, as detecting environmental information relative to the performer (e.g., time-to-ground) enables perceiving the necessary movements to surpass the current situation. Previous research has demonstrated how detecting information about affordances enables the regulation of goal-directed movements, such as passing a ball in rugby [13], catching a flying ball in baseball [14], or walking in irregular terrain [15]. This shows how the perception of affordances and the control of movement are mutual and reciprocal [16].

By acknowledging the contextual and goal-directed nature of behavior, the ecological dynamics theoretical framework views performance as emerging and self-organizing under individual, environmental, and task constraints [10,17]. Different contexts influence the demands of sport and exercise tasks because environmental constraints play a critical role in how movement adapts from the interaction of multiple systems and subsystems (i.e., self-organizes). For example, indoor wall climbing versus outdoor bouldering climbing offer distinct affordances despite generally sharing movement patterns. The same applies to road cycling versus mountain biking, futsal versus beach football, and calisthenics in a gym versus in a park. Even if the rulesets of these sports and exercises are similar, the distinct environmental properties constrain different movement adaptations, along with distinct psychological effects, such as mood and attention [9]. That is, during sport and exercise tasks, the co-adaptation between performers and their environment results in more or less functional movement, accompanied by embodied and embedded psychological experiences [18].

To understand the emergence of movement and feeling (performance), an analysis of the typical constraints of nature-based and built environments is essential. Next, the typical constraints of outdoor nature-based and built indoor exercise environments are described in light of the action opportunities they offer.

## 3. Performing (Realizing Affordances) in Outdoor Nature-Based and Built Indoor Environments

Nature-based sport and exercise environments are outdoor settings in which natural elements constitute the dominant environmental characteristics. They encompass both natural ecosystems (e.g., forests, mountains, beaches, and rivers) and managed or semi-natural environments (e.g., urban parks, gardens, and green spaces). These environments contrast sharply with built indoor environments, which are enclosed, human-constructed spaces where environmental conditions (e.g., temperature, lighting, ventilation, and acoustics) are partially or fully controlled (e.g., gymnasiums, fitness centers, and indoor sports facilities). These environments generally represent the most distant counterparts in terms of the ratio of natural-to-built features, so this article focuses on these two contrasting environments.

**Figure 1:**
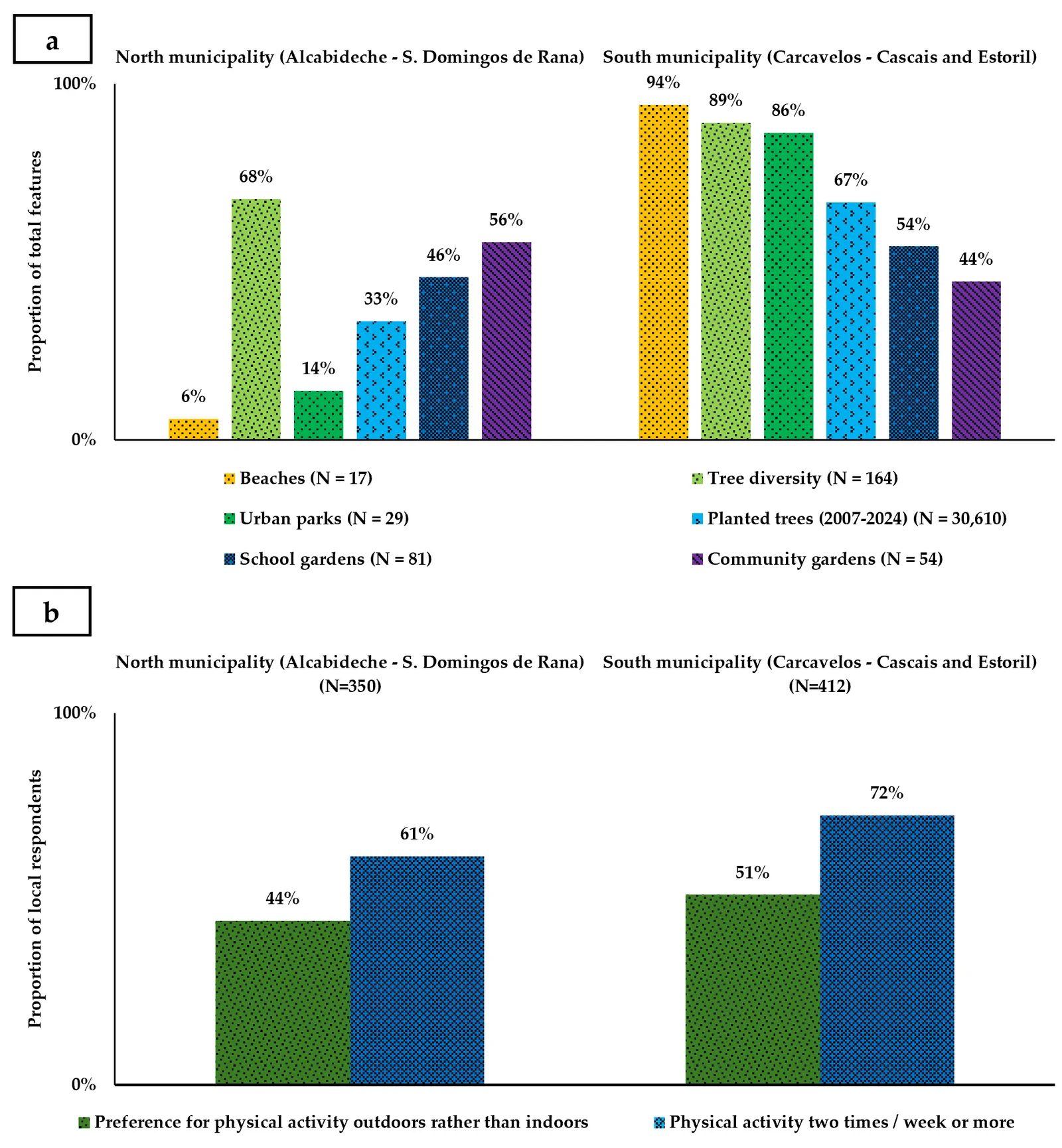
(**a**) Descriptive differences in nature-based features and physical activity in the north and south municipalities of Cascais, Portugal. The tree species variable represents species richness, which may be shared between municipalities, unlike the other variables, which represent unique physical locations. The reported sample size in (**b**) refers to the survey respondents included in the original municipal dataset for 2023–2024, compiled from public databases of the Cascais municipality services, Portugal, available at https://data.cascais.pt/ (accessed on 14 August 2025).

Epidemiological evidence on urban development and ecology shows that, at the city level, having larger urban green spaces, such as parks and tree-lined avenues, affords more physical activity [19,20]. The unique constraints of large, open, nature-based environments channel and afford more or less sport and exercise behavior, because when there is more green space, city dwellers can cover a larger area without motorized transportation, allowing them to be more active for longer [21,22]. Figure 1 illustrates how the south municipality of Cascais, Portugal, featured more nature-based environments than the north municipality (a) and shows that the population in the south was more physically active and preferred outdoor activity (b).

The purpose of Figure 1 is not to establish a causal relationship between environmental characteristics and physical activity, but to provide descriptive support for the well-established premise that places with greater availability of natural outdoor environments usually exhibit higher levels of physical activity in the local population [19,21].

Naturally, affordances exist in both nature-based and built indoor environments, but their availability is tied to the constraints of the environment [23]. In nature, exercise occurs in rich, varied, dynamic conditions. These include variable features such as wind, rain, scents, sunlight, temperature, obstacles (e.g., holes, logs, bushes), uneven terrain, and diverse textures (e.g., grass, rocks, wood) [23]. Conditions can shift suddenly (e.g., it can start to rain) or the performer may need to transition from a trimmed lawn to dense brush. Biodiversity in nature-based environments is often visible and audible, making it an inherent part of the sport and exercise experience. Nature-based environments are also known to afford contemplative and relaxing behavior, which supports well-being. Vainio et al. [24] detailed how specific types of trees afford nostalgic, nurturing, and empowering human-arboreal relations. This aligns with Heft’s [23] account that nature provides a “richness of information available to be perceived” (p. 245). Conversely, built indoor exercise environments are less variable and standardized; hence, they become expected. Designed indoor spaces, such as game courts, pitches, and gyms, feature smooth and unobstructed surfaces, climate-controlled conditions, and game/exercise area sizes that are similar from facility to facility. In particular, training machines in gym facilities often restrict movement to predetermined amplitudes and directions, thereby limiting exploration. It is possible to introduce environmental variability, for example, by crowding the environment (team sports with many players tend to be complex, dynamic environments), but here we suggest that similar tasks, such as team sports, might still be influenced by the elevated complexity and dynamics of the inherent variable constraints of nature-based environments, even in manicured nature-based environments (e.g., wind variations in urban parks). Hence, the standardized and controlled conditions in built indoor environments may not require as much movement adaptability, eliminating the need for sudden accommodations. This, in turn, may reinforce repetitive patterns, reduce the need for affordance perception, and result in monotonous action and boredom. Overall, constraints of nature-based and built indoor environments can shape how performers couple perception and action during physical activity in different ways [5].

Moreover, a comprehensive description of performer–environment interactions includes the role of sociocultural constraints. Specifically, designed indoor environments can be shaped by strict sociocultural norms, influencing how instruments and spaces are used (e.g., the “correct” way to use gym equipment), which may limit creative solution-finding [25]. The stability of built indoor environments can have obvious advantages, but it also can lead to disengagement, with performers focusing on unrelated thoughts rather than the task at hand. In contrast, the variability usually found in nature-based environments can be expected to require full-body perception and action to be tightly coupled and task-oriented. Crucially, interacting with public green space can also be shaped by sociocultural constraints, such as public policies that prohibit stepping onto the lawn or climbing on top of trees. However, there is an essential nuance of behaving among these environments when compared to built indoor settings, because natural features like trees, logs, and rocks are not designed for specific actions. Instead, they can offer an open-ended landscape of affordances for movement exploration according to each performer’s skills, characteristics, and goals, which can foster adaptability and engagement. Next, we demonstrate how performers adapt their movement patterns to the specific sport and exercise environment.

## 4. Evidence of Skill Adaptation in Nature-Based Sport and Exercise

**Figure 2:**
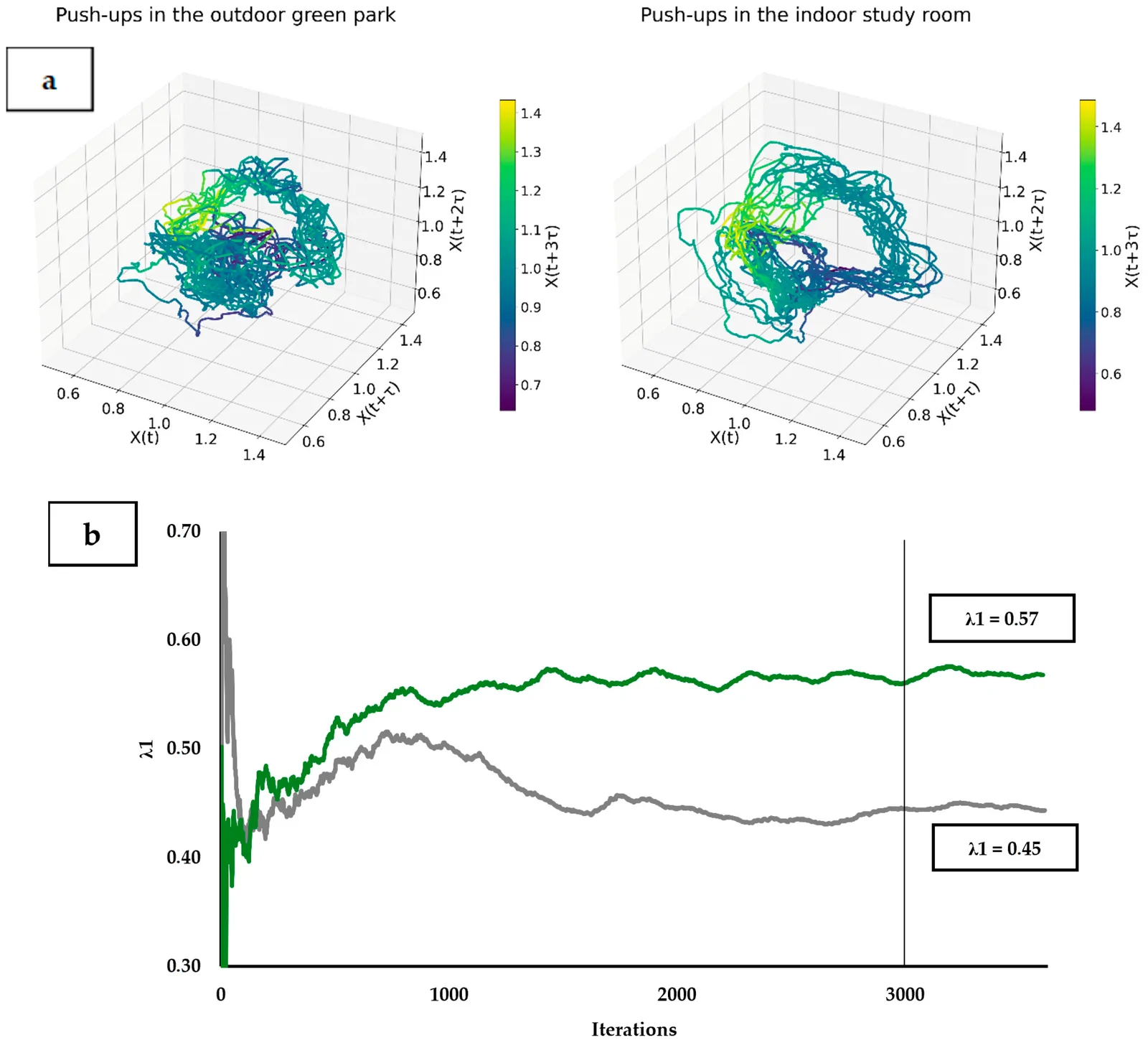
State space trajectories of performing push-ups in a green park vs. a study room. The state space (**a**) shows a time series (X(*τ*)) of antero-posterior (frontal plane) accelerations (g) of the lower back during push-ups, with *τ* as the lag of the time series, as defined by the Average Mutual Information method. False Nearest Neighbors method was also used to define the dimension *n*, which turned out to be 4. The bottom plot (**b**) shows a running average of Largest Lyapunov Exponents (λ1)—a statistical algorithm that quantifies how rapidly nearby trajectories diverge in a nonlinear dynamical system—plotted against evolution iterations (following the algorithm of Wolf et al. [27]). The green line refers to λ1 in the nature-based setting and the gray line refers to λ1 in the built indoor setting. Final λ1 values were established by the steady state after the 3000th iteration marked in the plot (as per Wurdeman [28]), represented in (**b**) as a black vertical line. Raw data from Brito et al. [5] were reused for the estimation of (λ1), with permission from the authors. Full methodological details of data collection (sensor placement, sampling frequency, task protocol) are reported in the original study [5], and raw data are publicly available at https://doi.org/10.17632/vyw4fdt8cb.2 (published on 21 July 2026).

Skill adaptation, defined as the reorganization of coordination and control over time based on affordance perception and action, enables performers to pursue task goals amid environmental changes [26]. Brito et al. [5] showed that participants who exercised once, either in an outdoor nature-based environment or a built indoor environment, required specific movement patterns to achieve the same goal of performing push-ups. The increased challenge of diverse circumstances in the nature-based setting required performers to have greater attunement to more variable affordances, consequently adapting their skills to achieve their goals. Figure 2 illustrates data from two representative participants (one exercising in a nature-based setting, one in a built indoor setting), selected from the dataset of Brito et al. [5]. The figure is presented as an illustration of how movement variability can differ between nature-based and built indoor environments, captured by distinct state space trajectories (greater Largest Lyapunov Exponent). In this case, higher values indicate greater movement variability, which may reflect exploration of different trajectories (solutions) by releasing degrees of freedom in order to achieve successive push-up repetitions.

Increased movement variability has been associated with higher-level performance levels, aligning with previous research. Mehdizadeh et al. [29] showed how movement variability could help differentiate between skilled and unskilled athletes in a shuttle agility drill. Robalo et al. [30] limited visual and auditory information during a basketball dribbling task in skilled and amateur basketball players. Even with information occlusion, skilled professional basketball players showed greater movement variability in shoulder horizontal movements and lateral elbow movements, which was associated with greater task success than that of amateurs. Furthermore, da Costa et al. [31] provided evidence that movement variability can serve as a marker of expertise, at least in simple tasks such as continuous clapping, walking, and upper-extremity movements (see also Seifert et al. [32]). Additionally, there is evidence that perturbations during physical tasks demand more control from performers. For example, Munoz-Martel et al. [33] showed that participants switching from a double- to a single-leg stand on an unstable oscillating platform required an additional muscle synergy to balance, compared to stable ground.

Task constraints, such as reducing the time to complete the task or imposing speed constraints, can increase variability. For example, Mehdizadeh et al. [34] noted that increasing running speed increased the variability of successive steps. However, successful performance in sport and exercise does not always require elevated levels of variability. The best performers can accommodate movement efficiently to changes in the environment, not varying movement unnecessarily (i.e., beyond an inefficiency threshold). Movement systems can become extremely variable or extremely stable, depending on the demands of the task-environment [11]. This means that movement variability is deeply situated and intertwined with environmental conditions, since perceiving affordances reflects how environmental features relate to a performer, while accounting for that performer’s ability to generate movement. In nature-based sport and exercise, environmental conditions are dynamic, increasing the complexity of the task, demanding increased attunement to relevant information sources and movement adaptability [5]. In other words, sport and exercise performers are required to sustain goal-directed movement that is both flexible and stable through the coupling of perception and action. In nature-based environments, performers are constrained to develop innovative solutions, shaped by their environment yet guided by their own skills [9]. Ultimately, variable but directed movement is necessary to be as effective as possible in sport and exercise, with skill adaptation being promoted by performing in uncertain task environments, such as natural settings.

However, there is a lack of research on longitudinal changes in skill adaptation for sport performance when engaging in nature-based environments (e.g., adventure sports). Examining these changes is most popular in the biomechanical, clinical, and rehabilitation fields [1]. However, the lack of evidence on the effects of nature-based environments for skill adaptation, health, and well-being in sport led us to the following question: how would prolonged, sustained sport and exercise in nature-based settings over developmentally meaningful timescales (weeks to years) affect athletes’ skills, health, and well-being? Next, a hypothesis is raised.

## 5. Hypothesis: Sustained Nature-Based Exercise Transforms Athletes’ Ability to Perceive and Act

The ecological dynamics perspective suggests that performers gradually attune to relevant sources of information by frequently realizing affordances in similar task-environment circumstances. Hence, performers learn to calibrate movement more efficiently over time under similar conditions [26].

The information flow created by the continuous relation between performers and the rugged, irregular challenges of nature-based environments may enable performers to exploit inherent movement self-organizing tendencies and develop skills under the dynamic constraints of nature-based environments over time [10]. Although nature-based exercise may initially require high (functional) variability (task-relevant and adaptive) of movement due to task-environment constraints, with experience, skilled performance is expected to become related to reduced variability and minimal stochastic noise. These changes toward adaptable stability in the structure of variability throughout the learning process have been described extensively [35,36]. However, as mentioned in Section 4, longitudinal studies examining movement adaptations to nature-based sport and exercise are lacking. Still, there is evidence that walking on uneven terrain, a staple of nature-based environments, induced follow-up changes in human walking dynamics [37]. While initial exposure may increase variability in gait parameters, as well as increased muscle activity, ongoing engagement can possibly lead to reduced variability, suggesting that the body adapts to the challenges posed by uneven surfaces; yet some level of variability will persist [38]. As a result of prolonged engagement, perhaps the performer can maintain a degree of responsiveness to general environmental changes.

**Figure 3:**
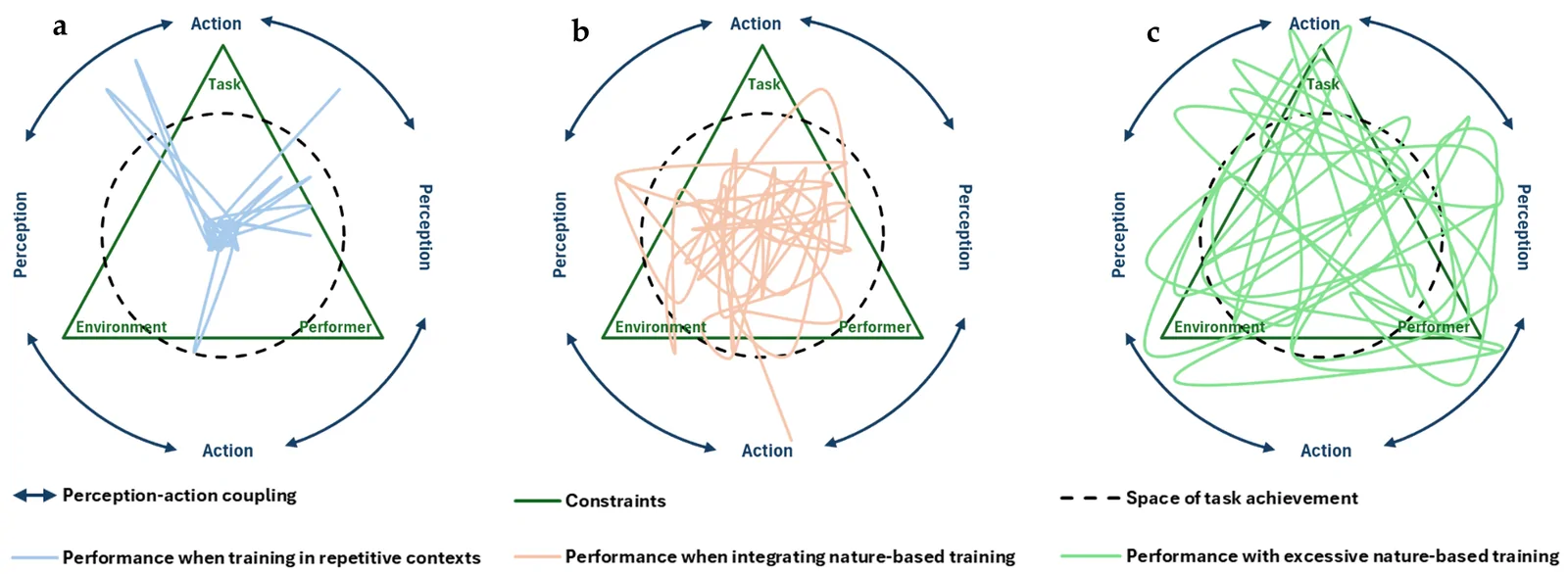
Hypothesized sport performance trajectories inside a space of task achievement, according to different levels of nature-based training integration into usual sport training, emerging from interacting performer, task, and environment constraints, and sustained by performers’ perception–action coupling. (**a**) Sport and exercise training in the usual environment (gym, sport facility, pitch) reinforces performers’ task solution-finding to a limited set of movement solutions; (**b**) integrating nature-based exercise into usual sport training supports solution-finding due to adaptation to inherent variability in nature-based settings, possibly transferring into performance in the usual sport and exercise facilities; (**c**) excessive nature-based training/exercise does not support task achievement in the usual sport and exercise facilities due to increased detachment with the usual environment characteristics.

Building on the previous point, it can be hypothesized that athletes who continually engage with nature-based environments during sport and exercise will adapt to contextual diversity. That is, they will develop the skill to shift from current, repetitive movement trajectories to more functionally adaptive ones [5]. The notion of skill adaptation in sport and exercise permits the assumption that, from these transactions with nature, athletes would benefit from bodily adaptations to act in other environmental contexts (e.g., indoors) [7,26]. Parkour is a primary example of skill adaptation, as it is an activity inspired by overcoming natural obstacles, such as rocks, trees, and rivers, which are staples of adventure sports like trail running and bouldering/climbing. Nevertheless, in competitive sport, the incorporation of nature-based training should be carefully planned alongside other more representative forms of training, as excessive detachment from the environments in which sport performance is to take place (regular pitch/court/field) may contribute to a loss of attunement and calibration to key properties of the usual competition environment, as is illustrated in Figure 3.

Importantly, the proposed integration of nature-based environments into sport training should not be interpreted as universally beneficial or appropriate across all contexts. The effectiveness of nature-based training is expected to depend on the interaction between performer, task, and environmental constraints, including athlete experience, sport-specific demands, accessibility, environmental conditions, safety, and the intended training outcomes. Nature-based environments may also introduce challenges, such as increased injury risk associated with unfamiliar or irregular terrain, adverse weather conditions, or limited accessibility for some athletes. Consequently, the inclusion of nature-based training should be individualized and designed to complement, rather than replace, representative sport-specific practice.

Performance entails not only context-dependent actions but also other psychological aspects. How athletes think and feel influences how skill adaptation unfolds, with emotional experiences shaping how they approach new tasks, which in turn modify the ongoing emotion–cognition–action relation [18]. Engaging in nature-based exercise not only can shape immediate psychological states but also may contribute to skill learning. Next, the benefits of nature-based exercise are discussed from a health and well-being perspective.

## 6. Nature-Based Environments: A Locus of Active Health and Well-Being

Training is essential for skill adaptation and performance. Crucially, for training to be successful, non-obvious and sometimes counterintuitive aspects and experiences are necessary to sustain and improve performance [39]. In particular, athletes’ mental health and well-being are necessary aspects and experiences for performance in training and competition to emerge. In this sense, the beneficial psychological outcomes of being active in nature can facilitate these necessary processes for performance. Evidence shows that engaging in nature-based sport and exercise provides greater well-being, more so than exercising in built indoor environments [4] and built urban environments [40]. According to Li et al. [41], more unmanicured green spaces are associated with greater benefits for health and well-being. Benefits of nature-based exercise include greater feelings of humility, courage, resilience, self-worth, and self-control; greater perceived affect and energy; and lower levels of perceived stress, depression, and anxiety [42]. Perceptions of greater affect and mood, as well as increased attention and concentration, may lead to lower stress and tension, allowing both recreational and expert performers to achieve superior performance [4].

Psychological theories suggest how interactions with nature-based environments facilitate well-being. Cognitive-behaviorist theoretical frameworks mainly focus on the inner motives, beliefs, and predetermined tendencies to act in these environments [43,44]. Thus, within these frameworks, nature-based environments are viewed as a source of beneficial stimuli, and exposure to nature is thought to facilitate cognitive functioning and mood enhancement mediated by personal factors [9,45]. Kaplan’s Attention Restoration Theory [43] and Ulrich’s Stress Reduction Theory [44] are examples of frameworks that propose that nature-based environments possess great properties of calmness induction, stress relief, and recovery of cognitive resources. However, these frameworks assume that nature experiences are objectively relaxing, which may not be the case for every individual or context (e.g., someone with arachnophobia or someone who undertakes adventure sports). As a result, in cognitive-behaviorist frameworks, beneficial stimuli are deemed identical for all, from passive bystanders to sport and exercise performers, perhaps mediated by personal preference and other internal factors. What distinguishes these frameworks from a perception–action understanding is that they do not consider the essential role of acting in the environment [9,46]. To highlight the importance of situated action, recent studies have demonstrated that participants engaging in a nature-based exercise program experienced enhanced self-perceptions of being active and well-being compared to those who received indoor behavior-change meetings aimed at encouraging constructive exercise adherence modifications [47,48]. It might also be that programmed nature-based experiences facilitate skill development for sport-related psychological issues such as performance anxiety [49,50].

As described in Section 2 and Section 3, the ecological dynamics theoretical framework considers nature-based exercise as occurring in settings with different affordances and, consequently, expressing different actions than in built environments [9]. Nature-based environments are predicated on the continuous evolution of the actual setting in previously unseen ways, requiring movement patterns to be constantly complemented with emerging, novel techniques and creative styles of action (e.g., bushwalking, trail running, skiing, whitewater kayaking). This striving clearly requires a continuous and intertwined relation between cognition, perception, and action [18], just as in built indoor settings, but in a distinct and nuanced way. Such non-anthropocentric, naturally variable features and phenomena in natural settings, including irregular surfaces and variable weather conditions, add to the challenge of nature-based sport and exercise. This facilitates, harnesses, and drives personal experiences, demanding a refined attunement to environmental information for skill adaptation and effective performance [51].

Emotions, feelings, and thoughts play a crucial role in regulating behavior within sport and exercise contexts. Any task-environment shapes emotional, feeling, and thought states, such as enjoyment, pain, or self-doubt, which emerge under interacting constraints. These, in turn, can influence how performers perceive and act upon affordances during practice. Previous research in taekwondo has demonstrated that distinct cognitive and affective states emerge within the training setting compared to competition, including decreases in perceived pressure, arousal, and mental challenges [52]. These findings emphasize the specificity of performer–environment interactions and highlight the importance of considering the emotional dynamics of competition to improve skill adaptation. This illustrates how emotion, cognition, perception, and action are interdependent processes that continually emerge through interactions between the performer and their environment [18]. Consequently, the constraints of nature-based environments can shape emotions, feelings, and thoughts in a way that promotes health and well-being in sport and exercise, beyond performance [4]. The ecological dynamics approach enables researchers, practitioners, and outdoors enthusiasts to understand how the experience of being active in nature is personally transformative and conducive to health, well-being, learning, and performance [9].

## 7. Discussion

### 7.1. Implementing Nature-Based Task-Environments in Sport

High-level sports involve full-time professional activity. Even at lower competition levels, training and related activities consume a significant portion of an athlete’s schedule. Multidisciplinary professionals, such as strength and conditioning coaches, nutritionists, performance analysts, and psychologists, strive to optimize this time to enhance performance, health, and well-being [53]. Here, we emphasize the hypothesized advantages of incorporating nature-based task-environments into athletes’ routines as complementary settings for training. These environments may afford rich perceptual–motor experiences alongside health and well-being benefits that ultimately support performance.

Engaging with variable and unanticipated environments potentially fosters skill adaptation by challenging athletes to regulate their actions in response to changing constraints [54]. For example, playing informal football on the beach requires athletes to detect varying time-to-contact information from irregular sand surfaces and adapt their actions to maintain coordination with the ball and the goal. The skills developed in these experiences—such as playing football at the beach—are thought to impact performance in competition by improving affordance perception and realization. Beyond performance, nature-based training may support and enhance psychological well-being, as discussed in Section 6. For instance, performing strength and conditioning sessions on the beach can simultaneously improve action capabilities while fostering enjoyment through direct interaction with natural elements, such as sand, sea, wind, and sun. Thus, health, well-being, and skill adaptation may act synergistically in nature-based task environments, as previously shown [5]. Moreover, nature-based activities can be integrated into season planning. In pre-season, they can help develop athletes’ structural conditioning, facilitate a transition back to routine by balancing leisure and training, and afford essential social connections (e.g., beach volleyball training sessions that combine practice and enjoyment with teammates). During the competitive period, they can serve as complementary or recovery sessions, helping to prevent training and action monotony that can lead to overuse injuries. In the off-season, they afford active rest and mental renewal while maintaining structural conditioning. In summary, coaches can plan training in natural conditions or design indoor tasks that represent the variability of affordances in nature to empower adaptive and healthy athletes [9].

### 7.2. Limitations

This review has limitations inherent to its narrative, hypothesis-generating nature. The literature was selected based on conceptual and empirical relevance rather than systematic review procedures, and the data presented in Figure 1 and Figure 2 are illustrative and exploratory rather than confirmatory. These limitations highlight the need for prospective research to test the proposed hypothesis, as longitudinal and direct evidence remain limited.

### 7.3. Future Directions

From an ecological dynamics perspective, a significant challenge in nature-based sport and exercise research is to identify the key constraints that impact the emergence of goal-directed movement. For example, what is it in built indoor and nature-based environments that has the potential to constrain behavior and promote skill adaptation? Environmental studies have focused on variables such as temperature, wind flow and direction, and ecosystem services; however, from an ecological dynamics perspective, as crucial as these aspects may be, they may not be the key information sources readily detectable to achieve task goals. For instance, inferring that athletes perform worse due to high environmental temperature attributes to temperature the guidance of behavior; rather, athletes regulate their goal-directed behavior based on the information detected in the environment. For example, for a white-water kayaker, how fast obstacles are approaching in the watercourse (e.g., rocks and boulders) informs what the necessary body, paddle, and kayak accommodations are needed to avoid crashing into them. Furthermore, how the water ahead looks, or the noise ahead, is relevant information for the kayaker to maintain control of the task at hand. It is this information-movement coupling that allows the kayaker to successfully navigate around such obstacles, denoting embodied, situated, and intelligent behavior [8]. This behavior can be captured and modeled by considering the distances and speed rates of the kayaker relative to task-relevant environmental properties, such as rocks and boulders, through the so-called eco-physical variables [55].

Eco-physical variables have emerged as a powerful tool to describe behavior in sport and exercise tasks [9,55]. These variables assess the fit between the athlete and the environment during goal-directed behavior, enabling experimental testing of the benefits of nature-based sport and exercise for skill adaptation, health, and well-being. In calisthenics, the ground surface used to support the hands during push-ups and squats, or the elevated bars used to hang during pull-ups, are key contact and target points, providing information that the athlete detects to continuously control their performance. Analyzing time-to-ground-contact during push-ups and squats, or time-to-bar-contact during pull-ups, may inform how nature-based environments influence the coordination dynamics of body segments compared with built indoor settings. Hence, eco-physical variables may express how athletes use environmental information to adapt their goal-directed movement. Moreover, these variables can benefit from nonlinear analysis, which provides insights into how athletes stabilize goal-directed behavior [55,56]. These dynamic processes are usually investigated using concepts related to stability and variability, which, although related, represent fundamentally different aspects. In particular, variability should not be uniformly equated with the stability or complexity of movement [57]. Therefore, movement variability directed toward stabilizing goal-directed behavior thus describes adaptive behavior [26], which nature-based training environments may harness.

Future research should test this hypothesis through longitudinal intervention studies that compare nature-based and conventional training, evaluate transfer to competitive performance, health and well-being, and skill adaptation, while controlling for training load. Future research should also determine the conditions under which nature-based training is safe, feasible, and beneficial across different sports, athlete populations, and environmental contexts.

## 8. Conclusions

In this article, we hypothesize that prolonged engagement in sport and exercise within nature-based environments promotes positive adaptations in skill development, health, and overall well-being in athletes, and that these benefits may subsequently enhance skill and performance when athletes return to indoor competitive settings. Nature-based and indoor sport and exercise environments present inherent environmental constraints that influence perception–action coupling. Built indoor settings typically require less adaptation due to stable conditions. In a nature-based environment, participants are required to adapt their movements to more variable circumstances when pursuing sport and exercise task goals, together with experiences of well-being. The demands and challenges of nature-based sport and exercise tasks can influence performance, health, and well-being in athletes, possibly enhancing adaptability in movement solutions. These enhancements to movement and well-being are based on an attuned perception–action coupling and may be stabilized by continuous, long-term engagement with nature-based features during sport and exercise tasks, and are adaptable to original (built indoor) sport and exercise settings such as sports arenas and facilities. Future longitudinal and experimental studies are needed to determine whether, and under what conditions, the strategic incorporation of nature-based activities into seasonal training plans can foster the proposed health, well-being, adaptive skill development, and performance-related benefits.

## Data Availability

Raw data used in this study are available online at: https://doi.org/10.17632/vyw4fdt8cb.2.

## References

[1] Ramachandran A.K., Pedley J.S., Moeskops S., Oliver J.L., Myer G.D., Lloyd R.S. (2024). Changes in Lower Limb Biomechanics Across Various Stages of Maturation and Implications for ACL Injury Risk in Female Athletes: A Systematic Review. Sports Med..

[2] Strachan L., Côté J., Deakin J. (2009). An Evaluation of Personal and Contextual Factors in Competitive Youth Sport. J. Appl. Sport Psychol..

[3] Steib S., Rettig-Hiebsch S., Schöttner H., Gabduliyanova A., Theil F., Wanner P., Köllner M.G. (2026). Implicit Power Motive, Affect, and Gross-Motor Skill Learning in a Contest Scenario. Perform. Enhanc. Health.

[4] Brito H., Carraça E.V., Palmeira A.L., Ferreira J.P., Vleck V., Araújo D. (2022). Benefits to Performance and Well-Being of Nature-Based Exercise: A Critical Systematic Review and Meta-Analysis. Environ. Sci. Technol..

[5] Brito H., Lopes H., Vaz de Carvalho M., Carrilho D., Carvalho A., Araújo D. (2024). The Effects of Nature-Based vs. Indoor Settings on the Adaptability, Performance and Affect of Calisthenics Exercisers. A Registered Report. Psychol. Sport Exerc..

[6] World Health Organization (2023). Assessing the Value of Urban Green and Blue Spaces for Health and Well-Being.

[7] Donnelly A.A., MacIntyre T.E., O’Sullivan N., Warrington G., Harrison A.J., Igou E.R., Jones M., Gidlow C., Brick N., Lahart I. (2016). Environmental Influences on Elite Sport Athletes Well Being: From Gold, Silver, and Bronze to Blue Green and Gold. Front. Psychol..

[8] Boobani B., Grants J., Glaskova-Kuzmina T., Zidens J., Litwiniuk A. (2024). Effect of Green Exercise on Stress Level, Mental Toughness, and Performance of Taekwondo Athletes. Arch. Budo.

[9] Araújo D., Brymer E., Brito H., Withagen R., Davids K. (2019). The Empowering Variability of Affordances of Nature: Why Do Exercisers Feel Better after Performing the Same Exercise in Natural Environments than in Indoor Environments?. Psychol. Sport Exerc..

[10] Button C., Seifert L., Chow J.Y., Araújo D., Davids K. (2021). Dynamics of Skill Acquisition: An Ecological Dynamics Approach.

[11] Davids K., Glazier P., Araújo D., Bartlett R. (2003). Movement Systems as Dynamical Systems: The Functional Role of Variability and Its Implications for Sports Medicine. Sports Med..

[12] Gibson J.J. (1979). The Ecological Approach to Visual Perception.

[13] Correia V., Araújo D., Craig C., Passos P. (2011). Prospective Information for Pass Decisional Behavior in Rugby Union. Hum. Mov. Sci..

[14] Fink P.W., Foo P.S., Warren W.H. (2009). Catching Fly Balls in Virtual Reality: A Critical Test of the Outfielder Problem. J. Vis..

[15] Matthis J.S., Yates J.L., Hayhoe M.M. (2018). Gaze and the Control of Foot Placement When Walking in Natural Terrain. Curr. Biol..

[16] Carello C., Wagman J.B., Sternad D. (2009). Mutuality in the Perception of Affordances and the Control of Movement. Progress in Motor Control: A Multidisciplinary Perspective.

[17] Newell K.M., Wade M.G., Whiting H.T.A. (1986). Constraints on the Development of Coordination. Motor Development in Children: Aspects of Coordination and Control.

[18] Araújo D., Davids K., Renshaw I., Tenenbaum G., Eklund R.C. (2020). Cognition, Emotion and Action in Sport: An Ecological Dynamics Perspective. Handbook of Sport Psychology.

[19] Nigg C., Fiedler J., Burchartz A., Reichert M., Niessner C., Woll A., Schipperijn J. (2024). Associations between Green Space Availability and Youth’s Physical Activity in Urban and Rural Areas across Germany. Landsc. Urban Plan..

[20] Chang P., Skov-Petersen H., Olafsson A.S. (2025). Exploring Multiscale Relationships between Environmental Characteristics and Recreational Trail-Based Activities in Urban Natural Areas: A Regional Study Leveraging User-Generated Big Data and Machine Learning. Urban For. Urban Green..

[21] Mao Y., Yin H., Bai Z., Yin J., Xia T., Wang L., Zhang J., Chen D. (2025). Walking, Jogging or Cycling? Exploring the Associations between Campus Greenway Environment and Physical Activity Using Large-Scale Trajectory Data. People Nat..

[22] Dietz L.W., Scepanovic S., Zhou K., Zanella A.F., Quercia D. (2025). Understanding the Potential of Urban Parks to Promote Well-Being. Nat. Cities.

[23] Heft H., Schutte A.R., Torquati J.C., Stevens J.R. (2021). Perceiving “Natural” Environments: An Ecological Perspective with Reflections on the Chapters. Nature and Psychology.

[24] Vainio K.K., Takala T., Limpens J., Lummaa K., Korrensalo A., Räsänen A., Tuittila E.-S. (2025). Green Companions: Affordances of Human–Tree Relationships. Ambio.

[25] Costall A. (2012). Canonical Affordances in Context. Avant.

[26] Araújo D., Davids K. (2011). What Exactly Is Acquired During Skill Acquisition?. J. Conscious. Stud..

[27] Wolf A., Swift J.B., Swinney H.L., Vastano J.A. (1985). Determining Lyapunov Exponents from a Time Series. Phys. D.

[28] Wurdeman S.R., Stergiou N. (2016). Lyapunov Exponent. Nonlinear Analysis for Human Movement Variability.

[29] Mehdizadeh S., Arshi A.R., Davids K. (2014). Quantification of Stability in an Agility Drill Using Linear and Nonlinear Measures of Variability. Acta Bioeng. Biomech..

[30] Robalo R.A.M., Diniz A.M.F.A., Fernandes O., Passos P.J.M. (2021). The Role of Variability in the Control of the Basketball Dribble under Different Perceptual Setups. Eur. J. Sport Sci..

[31] da Costa C.S.N., Batistão M.V., Rocha N.A.C.F. (2013). Quality and Structure of Variability in Children during Motor Development: A Systematic Review. Res. Dev. Disabil..

[32] Seifert L., Button C., Davids K. (2013). Key Properties of Expert Movement Systems in Sport. Sports Med..

[33] Munoz-Martel V., Santuz A., Ekizos A., Arampatzis A. (2019). Neuromuscular Organisation and Robustness of Postural Control in the Presence of Perturbations. Sci. Rep..

[34] Mehdizadeh S., Arshi A.R., Davids K. (2014). Effect of Speed on Local Dynamic Stability of Locomotion under Different Task Constraints in Running. Eur. J. Sport Sci..

[35] Guimarães A., Ugrinowitsch H., Dascal J., Porto A., Okazaki V. (2020). Freezing Degrees of Freedom During Motor Learning: A Systematic Review. Mot. Control.

[36] Müller H., Sternad D., Sternad D. (2009). Motor Learning: Changes in the Structure of Variability in a Redundant Task. Progress in Motor Control: A Multidisciplinary Perspective.

[37] Kent J.A., Sommerfeld J.H., Stergiou N. (2019). Changes in Human Walking Dynamics Induced by Uneven Terrain Are Reduced with Ongoing Exposure, but a Higher Variability Persists. Sci. Rep..

[38] Voloshina A.S., Ferris D.P. (2015). Biomechanics and Energetics of Running on Uneven Terrain. J. Exp. Biol..

[39] Turvey M.T., Sheya A. (2017). Non-Obvious Influences on Perception-Action Abilities. Psychon. Bull. Rev..

[40] Wicks C., Barton J., Orbell S., Andrews L. (2022). Psychological Benefits of Outdoor Physical Activity in Natural versus Urban Environments: A Systematic Review and Meta-Analysis of Experimental Studies. Appl. Psychol. Health Well-Being.

[41] Li H., Zhang X., Bi S., Cao Y., Zhang G. (2022). Psychological Benefits of Green Exercise in Wild or Urban Greenspaces: A Meta-Analysis of Controlled Trials. Urban For. Urban Green..

[42] Rosa C.D., Larson L.R., Collado S., Geiger S.J., Profice C.C., Menuchi M.R.T.P. (2025). Associations between Depression and Nature-Based Recreation: A Cross-Sectional Study of Adults in the United States, Spain, and Brazil. Sci. Rep..

[43] Kaplan S. (1995). The Restorative Benefits of Nature: Toward an Integrative Framework. J. Environ. Psychol..

[44] Ulrich R.S., Simons R.F., Losito B.D., Fiorito E., Miles M.A., Zelson M. (1991). Stress Recovery during Exposure to Natural and Urban Environments. J. Environ. Psychol..

[45] Houge Mackenzie S., Hodge K., Filep S. (2023). How Does Adventure Sport Tourism Enhance Well-Being? A Conceptual Model. Tour. Recreat. Res..

[46] Dzewaltowski D.A. (1997). The Ecology of Physical Activity and Sport: Merging Science and Practice. J. Appl. Sport Psychol..

[47] Chaves Dias C.R., Rocha S.V., Dias M.F., Morbeck de Queiroz B., Silva N.N., Conceição A.F., Palotino-Ferreira B.M., Brito-Costa S., de Lurdes Rosas da Silva S., Alves dos Santos C. (2025). Health Perceptions of Older Individuals on the Cognitive Benefits of a Multicomponent Green Physical Activity Program: A Qualitative Exploration in the Southwest Region of Bahia, Brazil. SSM Ment. Health.

[48] Marcen C., Cardona-Linares A.J., Pradas F., Ortega-Zayas M.A. (2024). Move to Flow: The Benefits and Barriers of a Physical Activity Nature-Based Pilot Programme. Sports.

[49] Baena-Extremera A., García J.F., Martínez A.C., Martín-Pérez C. (2021). Sports in Natural Environment, Sports in Urban Environment: An fMRI Study about Stress and Attention/Awareness. J. Sports Sci. Med..

[50] MacIntyre T., Nigg C., Murphy C., Oblinger-Peters V. (2023). Nature-Based Interventions in Elite Sport. Routledge Handbook of Mental Health in Elite Sport.

[51] Brymer E., Araújo D., Davids K., Pepping G.-J. (2020). Conceptualizing the Human Health Outcomes of Acting in Natural Environments: An Ecological Perspective. Front. Psychol..

[52] Maloney M.A., Renshaw I., Headrick J., Martin D.T., Farrow D. (2018). Taekwondo Fighting in Training Does Not Simulate the Affective and Cognitive Demands of Competition: Implications for Behavior and Transfer. Front. Psychol..

[53] Hauser L.-L., Höner O., O’Connor D., Wachsmuth S. (2025). Exploring the Interplay of Functional and Dysfunctional Environmental Features for Holistic Development: A Case Study in a German Olympic Sport. J. Appl. Sport Psychol..

[54] Caldeira P., Paulo A., Veloso A., Infante J., Davids K., Araújo D. (2024). How Functional Movement Variability Facilitates Successful Skill Adaptation during the Volleyball Attack. Int. J. Sports Sci. Coach..

[55] Lopes H., Carrilho D., Vaz de Carvalho M., Brito H., Carvalho A., Araújo D. (2025). The Role of Eco-Physical Variables for Analyzing and Modeling Goal-Directed Behavior in Sport. Acta Psychol..

[56] Stergiou N. (2016). Nonlinear Analysis for Human Movement Variability.

[57] van Emmerik R.E.A., Ducharme S.W., Amado A.C., Hamill J. (2016). Comparing Dynamical Systems Concepts and Techniques for Biomechanical Analysis. J. Sport Health Sci..

